# Successful electrohydraulic lithotripsy of an enterolith in Crohn’s disease using a small-caliber double-balloon endoscope

**DOI:** 10.1055/a-2904-8944

**Published:** 2026-07-14

**Authors:** Yuko Murashima, Ryosuke Horio, Yuki Ohta, Takashi Taida, Kenichiro Okimoto, Tomoaki Matsumura, Jun Kato

**Affiliations:** 1 Department of Gastroenterology 12737 Graduate School of Medicine, Chiba UniversityChibaChiba PrefectureJapan

**Fig. 1:**
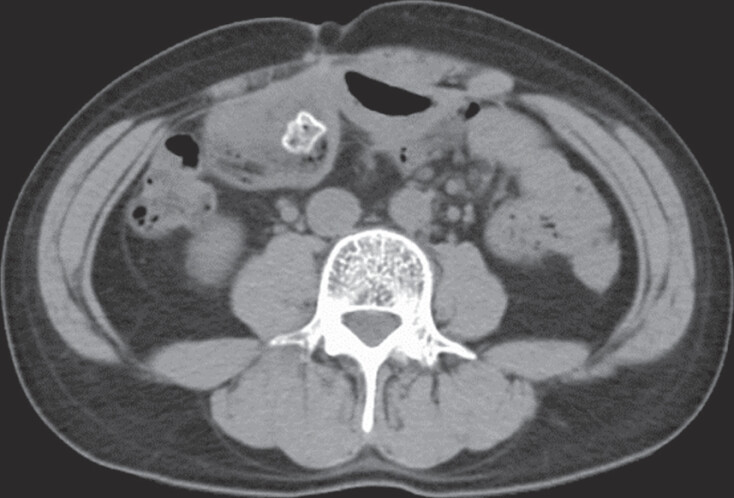
Computed tomography revealed a 25-mm high-density object in the dilated small bowel proximal to a stenosis.

A 54-year-old man with Crohn’s disease who had a history of small bowel resection was referred for treatment of an enterolith. The patient experienced abdominal pain and vomiting approximately once every few months. Computed tomography revealed small bowel stenosis with proximal prestenotic dilatation and a 25-mm high-density object within the dilated bowel lumen ( Fig. 1 ).

**Fig. 2:**
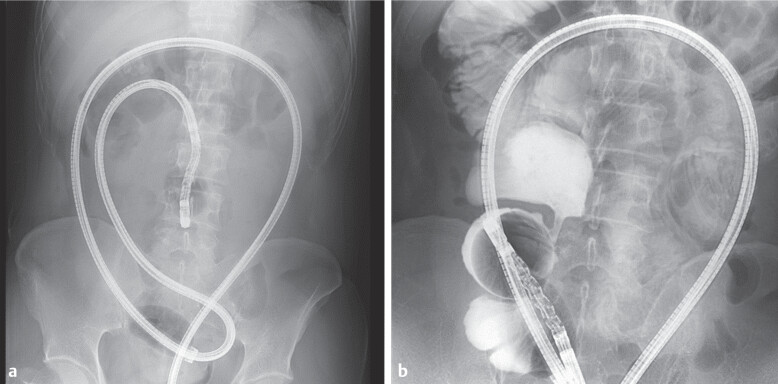
( **a** ) The endoscope was able to pass through the stenosis. ( **b** ) Retrograde contrast imaging during double-balloon endoscopy revealed a small bowel stenosis with a longitudinal ulcer 80 cm proximal to the ileocecal valve.

**Fig. 3:**
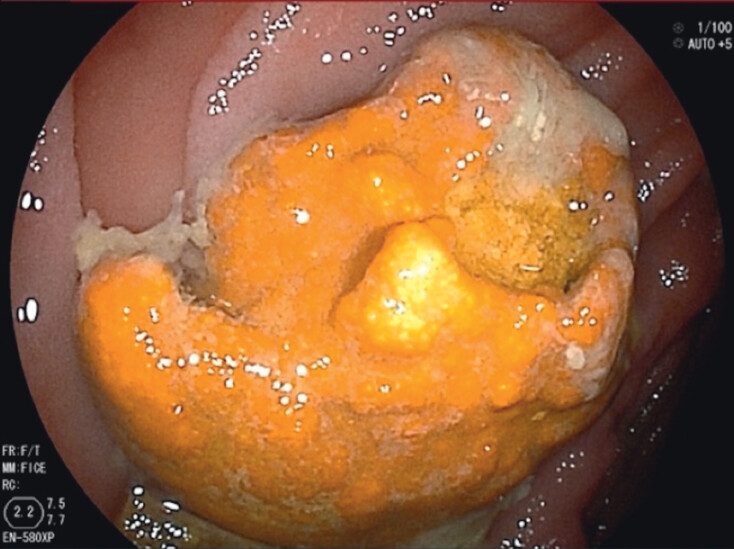
An enterolith was found within the dilated small bowel proximal to the stenosis.

**Fig. 4:**
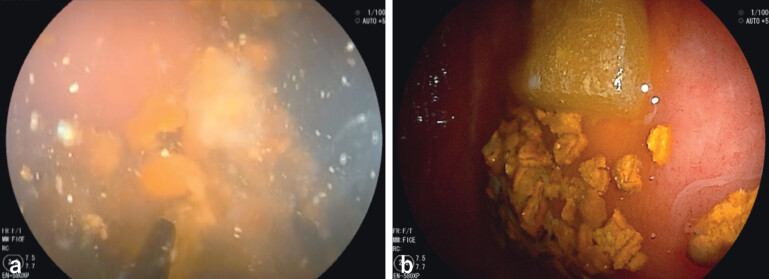
( **a** ) The enterolith was cracked using electrohydraulic lithotripsy. ( **b** ) The enterolith was fragmented into small pieces.

Because passage through the stenotic segment was anticipated to be difficult with a conventional therapeutic endoscope, we performed retrograde balloon-assisted enteroscopy using a small-caliber double-balloon endoscope (EN-580XP; Fujifilm, Tokyo, Japan; scope diameter 7.5 mm, working channel diameter 2.2 mm). A small bowel stenosis with a longitudinal ulcer, through which the small-caliber endoscope could barely pass, was identified 80 cm proximal to the ileocecal valve and an enterolith was found at the proximal side of the stenosis. Because the patient’s symptoms were likely to be caused by the enterolith, we decided to perform endoscopic fragmentation ( **Figs.** 2 and 3 ). Mechanical fragmentation with biopsy forceps was unsuccessful because of the hardness of the enterolith; therefore, we performed electrohydraulic lithotripsy (EHL; Video 1 ). The enterolith was successfully cracked and fragmented into small pieces without any adverse events ( Fig. 4 ). After the procedure, the patient became free from abdominal symptoms.

**Video 1** Successful electrohydraulic lithotripsy of an enterolith located proximal to a stenosis with an ulcer using a small-caliber double-balloon endoscope was performed.


Symptomatic enteroliths are usually treated with endoscopic mechanical lithotripsy. 1​ In this case, however, the therapeutic endoscope (e.g. EI-580BT; Fujifilm, Tokyo, Japan; scope diameter 9.4 mm, working channel diameter 3.2 mm), with which devices for mechanical lithotripsy are available, could not have passed through the stenosis and balloon dilatation would also have been difficult due to the presence of the ulcer at the stenosis. The device for EHL can work with the small-caliber scope with a narrower 2.2 mm channel and enabled successful enterolith fragmentation, demonstrating the feasibility of this approach in ulcer-associated stenosis 2​ ​3​ .

Endoscopy_UCTN_Code_TTT_1AP_2AD
